# Crestal Bone Loss and Peri‐Implant Conditions at Tissue‐Level Implants: Influence of Prosthesis Type After 25 Years

**DOI:** 10.1111/cid.70158

**Published:** 2026-05-14

**Authors:** Clemens Raabe, Manrique Fonseca, Andrea Roccuzzo, Martin Schimmel, Giovanni E. Salvi, Vivianne Chappuis, Emilio Couso‐Queiruga

**Affiliations:** ^1^ Department of Oral Surgery and Stomatology University of Bern School of Dental Medicine Bern Switzerland; ^2^ Department of Oral Surgery and Implantology Goethe University Frankfurt am Main Germany; ^3^ Department of Reconstructive Dentistry and Gerodontology University of Bern School of Dental Medicine Bern Switzerland; ^4^ Shanghai Perio‐Implant Innovation Center, Institute of Integrated Oral, Craniofacial and Sensory Research, Shanghai Ninth People's Hospital, Shanghai Jiao Tong University School of Medicine. College of Stomatology, Shanghai Jiao Tong University, National Center for Stomatology, National Clinical Research Center for Oral Diseases, Shanghai Key Laboratory of Stomatology, Shanghai Research Institute of Stomatology Shanghai China; ^5^ Department of Periodontology University of Bern School of Dental Medicine Bern Switzerland

**Keywords:** bone resorption, clinical trial, dental implants, guided bone regeneration, peri‐implantitis, phenotype

## Abstract

**Objective:**

To evaluate long‐term crestal bone level changes (ΔCBL) and prevalence of peri‐implant conditions according to the type of implant‐supported fixed dental prosthesis (IFDP), and to identify implant‐, prosthetic‐, and patient‐related factors associated with ΔCBL.

**Materials and Methods:**

This single‐center observational study evaluated patients rehabilitated with single crowns (SCs), splinted crowns (SPs), or bridges (BRs) on tissue‐level implants after 10 and 25 years of function. Standardized clinical and radiographic evaluations were performed, and peri‐implant conditions were diagnosed according to established consensus definitions.

**Results:**

A total of 147 patients with 233 implants were included. The prevalence of peri‐implantitis (PI) increased from 10 to 25 years, from 2.3% to 7.8% for SC, from 1.6% to 6.3% for SP, and from 0% to 4.9% for BR, with no significant differences between IFDP types at either time point (*p* ≥ 0.95). SP tended to have higher peri‐implant mucositis (PM) rates at 25 years (87.3%), compared to SC (74.4%) and BR (80.5%). Mean ΔCBL was −0.37 ± 0.91 mm for SC, −0.25 ± 0.85 mm for SP, and −0.70 ± 1.0 mm for BR, with no significant differences between IFDP types (*p* = 0.37). Multivariate analyses demonstrated that greater ΔCBL was significantly associated with incisor region location (−0.74 mm), increased probing depth (−0.16 mm per mm), and suppuration on probing (−0.97 mm), all *p* ≤ 0.05. In contrast, prosthetic retention, marginal misfit, and prosthet emergence geometry were not significantly associated with ΔCBL.

**Conclusions:**

Over 25 years, the type of IFDP did not significantly affect ΔCBL or the prevalence of PM and PI around tissue‐level implants. Long‐term bone stability was primarily influenced by implant location in the incisor region, increased probing depths, and the presence of suppuration.

## 1 Introduction

Dental implants have evolved into a predictable and widely accepted treatment modality for the rehabilitation of partially and completely edentulous patients across all age groups [1, 2]. Long‐term success of implant therapy depends on multiple interrelated factors, including appropriate patient selection, comprehensive treatment planning, precise surgical execution, suitable implant and prosthetic design, and consistent supportive care aimed at preserving peri‐implant tissue health over time [1]. A key indicator of peri‐implant health (PH) is the stability of the crestal bone levels (CBL) following the initial phase of physiological remodeling that generally occurs after implant placement and functional loading of the implant‐supported fixed dental prosthesis (IFDP) [3, 4, 5]. In contrast, progressive bone loss compromises the structural and biological integrity of the implant and increases the risk of implant failure through both infectious and mechanical pathways [5, 6, 7, 8].

Dental implants may support removable dental prostheses, or IFDPs, such as single crowns (SC), splinted crowns (SP), or bridges (BR). Evidence indicates that IFDP type and prosthetic characteristics could have an influence on PH and CBL. Greater bone loss and a higher prevalence of peri‐implantitis (PI) have been reported for splinted compared with nonsplinted IFDPs, in the presence of marginal misfit or interproximal contact loss [9, 10, 11, 12, 13, 14]. In contrast, the type of prosthetic retention, such as screw‐retained or cement‐retained, does not appear to substantially influence PI prevalence or CBL. However, residual excess cement remains a critical local factor that may predispose the onset and progression of peri‐implantitis [12, 15, 16, 17, 18, 19].

Recent evidence further highlights the influence of implant design and prosthetic emergence geometry parameters on peri‐implant tissue stability. Emergence angles exceeding 30° and convex emergence profiles have been consistently associated with greater CBL and an increased risk of PI, particularly in bone‐level implants [20, 21, 22]. Moreover, wider implant diameters have been significantly correlated to a higher prevalence of peri‐implantitis, whereas taller prosthetic platform height has been associated with reduced CBL [23, 24]. Nevertheless, the long‐term effect of IFDP type and associated transmucosal emergence geometry in tissue‐level implants on peri‐implant diseases and changes in CBL over time (ΔCBL) remains largely underexplored.

Therefore, the primary objective of the present study was to assess the long‐term ΔCBL according to IFDP type at tissue‐level implants. Secondary objectives were to (1) evaluate the prevalence of peri‐implant conditions, (2) determine the influence of transmucosal emergence geometry, as well as implant‐, prosthetic‐, and patient‐ related factors on ΔCBL, and (3) characterize peri‐implant bone‐related radiographic findings.

## 2 Materials and Methods

### 2.1 Study Design, Setting, and Ethical Approval

This single‐center observational study was conducted in accordance with the Strengthening the Reporting of Observational Studies in Epidemiology (STROBE) guidelines and the principles of the Declaration of Helsinki (1975), as revised in 2024 [25, 26]. Ethical approval was obtained from the Cantonal Ethics Committee of Bern, Switzerland (KEK‐BE‐No. 2023‐02279), and the study was prospectively registered at ClinicalTrials.gov (NCT06599320). Clinical and radiographic data were collected at the Department of Oral Surgery and Stomatology, School of Dental Medicine, University of Bern, Switzerland, during two periods: January 2010 to January 2011 and September 2024 to May 2025.

### 2.2 Eligibility Criteria and Recruitment

Health records of adult patients who underwent tooth replacement therapy with IFDPs between May 1997 and January 2001 were reviewed for eligibility [27]. Inclusion criteria were: (1) age ≥ 18 years; (2) partial and complete edentulism; (3) presence of at least one tissue‐level titanium implant with a sandblasted and acid‐etched surface for the replacement of a missing tooth; and (4) restoration with a cement‐ or screw‐retained IFDP. Exclusion criteria included: (1) insufficient clinical or radiographic documentation (e.g., of failed implants); (2) refusal to undergo further clinical or radiographic examinations; and (3) presence of conditions that impair the comprehension, reading, or signing of informed consent. Eligible patients were invited to attend clinical and radiographic follow‐up examinations by telephone. Written informed consent was obtained from all participants after a detailed explanation of the study objectives and procedures, with opportunities to ask questions. Finally, for inclusion in the present investigation, patients were required to have participated in both clinical and radiographic follow‐up examinations; patients lacking either assessment were excluded from the analysis.

### 2.3 Variables of Interest

#### 2.3.1 Primary Outcome

To evaluate the impact of IFDP type on ΔCBL.

#### 2.3.2 Secondary Outcomes

To assess (1) the prevalence of peri‐implant conditions (peri‐implant mucositis (PM) and PI), as defined by the World Workshop on Periodontal and Peri‐Implant Diseases and Conditions [28] and in accordance with the EFP S3‐level clinical practice guideline on the prevention and treatment of peri‐implant diseases [29], (2) the influence of transmucosal emergence geometry, as well as patient‐ and prosthesis‐related factors on ΔCBL, and (3) to record other radiographic findings.

### 2.4 Clinical Data Collection

At the 25‐year follow‐up, comprehensive oral examinations were performed by two calibrated periodontists (A.R. and E.C.‐Q.). Calibration included a protocol review and joint assessment of five randomly selected patients to ensure methodological consistency. Clinical measurements were recorded using a UNC‐15 periodontal probe (Hu‐Friedy, Chicago, IL, USA) and included keratinized mucosa width (KMW), direct measurement of the facial mucosal thickness 3 mm apical to the mucosal zenith (FMT), probing depth (PD), bleeding on probing (BOP), and suppuration on probing (SOP) at six sites per implant. The type of IFDP was documented as: (1) SC; (2) SP, with or without cantilever; (3) BR, with or without cantilever, whereas non‐IFDP included (4) tooth‐implant supported bridges, or (5) removable dental prostheses. The type of IFDP retention (screw‐ vs. cement retained) was recorded. Patient demographics, medical and dental histories, surgical and prosthetic variables, participation in supportive peri‐implant care, and implant location were retrieved from health records and structured questionnaires. Results of the 10‐year follow‐up evaluation have been previously reported [27].

### 2.5 Radiographic Data Collection

For diagnostic evaluation, identical radiographic methods were used at both the 10‐ and 25‐year follow‐ups. Periapical radiographs (Xios XG Supreme, Size 2; Dentsply Sirona, Charlotte, NC, USA) were obtained using the parallel technique with standardized film holders (XCP, Dentsply Sirona), and panoramic radiographs were acquired (Veraview X800, Morita Corp., Osaka, Japan). Two independent, calibrated examiners (C.R. and E.C.‐Q.) assessed all radiographs using open‐source software (ImageJ, National Institutes of Health [NIH], Bethesda, MD, USA). Calibration was performed using the first 15 consecutive images. The known thread pitch distance of each implant was used to convert pixel values to millimeters. To minimize angulation effects, CBL and prosthetic emergence geometry were assessed on panoramic radiographs using a horizontal line drawn at the implant platform level as a reference. Perpendicular vertical lines were drawn to the first radiographically visible bone‐to‐implant contact at the mesial and distal aspects [30]. The respective implant platform height was then subtracted from these distances to calculate the crestal bone level (CBL). If individual measurements differed by more than 0.2 mm, both examiners re‐evaluated the image and reached consensus on the precise bone level. The mean of both examiners' final values was used for analysis. The crown emergence angle was defined as the angle between the implant long axis and a tangent to the restoration at the level of the implant platform, and the emergence profile was classified according to an established methodology [20]. The presence of IFDP‐misfit and peri‐implant bone‐related radiographic findings was assessed dichotomously on periapical radiographs [10, 31].

### 2.6 Statistical Analysis

All analyses were performed using the statistical software R, version 4.4.2 [32]. Demographic, implant‐, and IFDP‐related characteristics, as well as clinical and radiographic outcomes, were stratified by IFDP type (SC, SP, and BR) and by peri‐implant diagnoses and descriptively summarized as means, medians, and standard deviations for continuous variables, and as frequencies and percentages for categorical variables. Intraclass correlation coefficients (ICCs) were calculated to assess inter‐rater agreement for CBL at the 10‐ and 25‐year follow‐up, as well as for IFDP emergence geometry at the 25‐year follow‐up. Differences in outcomes between the IFDP types were tested using robust linear mixed regression models and a multinomial‐Poisson generalized linear mixed model (GLMM) for nominal response data, with patient ID included as a random effect. Model goodness‐of‐fit was assessed by inspection of residuals.

Robust linear mixed regression models were also used to assess factors associated with ΔCBL, defined as the change in CBL between the 10‐ and the 25‐year follow‐ups. To avoid overparameterization, factors were first screened in univariate models and included in the final multivariate model if the *p*‐value was ≤ 0.10 and if they were not correlated with other factors. Goodness‐of‐fit of the final model was assessed by inspection of residuals and testing for collinearity, with location (mesial vs. distal) included as an additional random (nuisance) effect.

Finally, the association between clinical and radiographic outcomes, including variables such as BOP, PD, and ΔCBL, was assessed using robust linear mixed regression models, adjusting for risk factors identified in the multivariate ΔCBL model. For all models, the observations‐to‐estimator ratio was at least 472/20 = 23.6. *p*‐values ≤ 0.05 were considered statistically significant.

## 3 Results

### 3.1 Study Cohort

**FIGURE 1:**
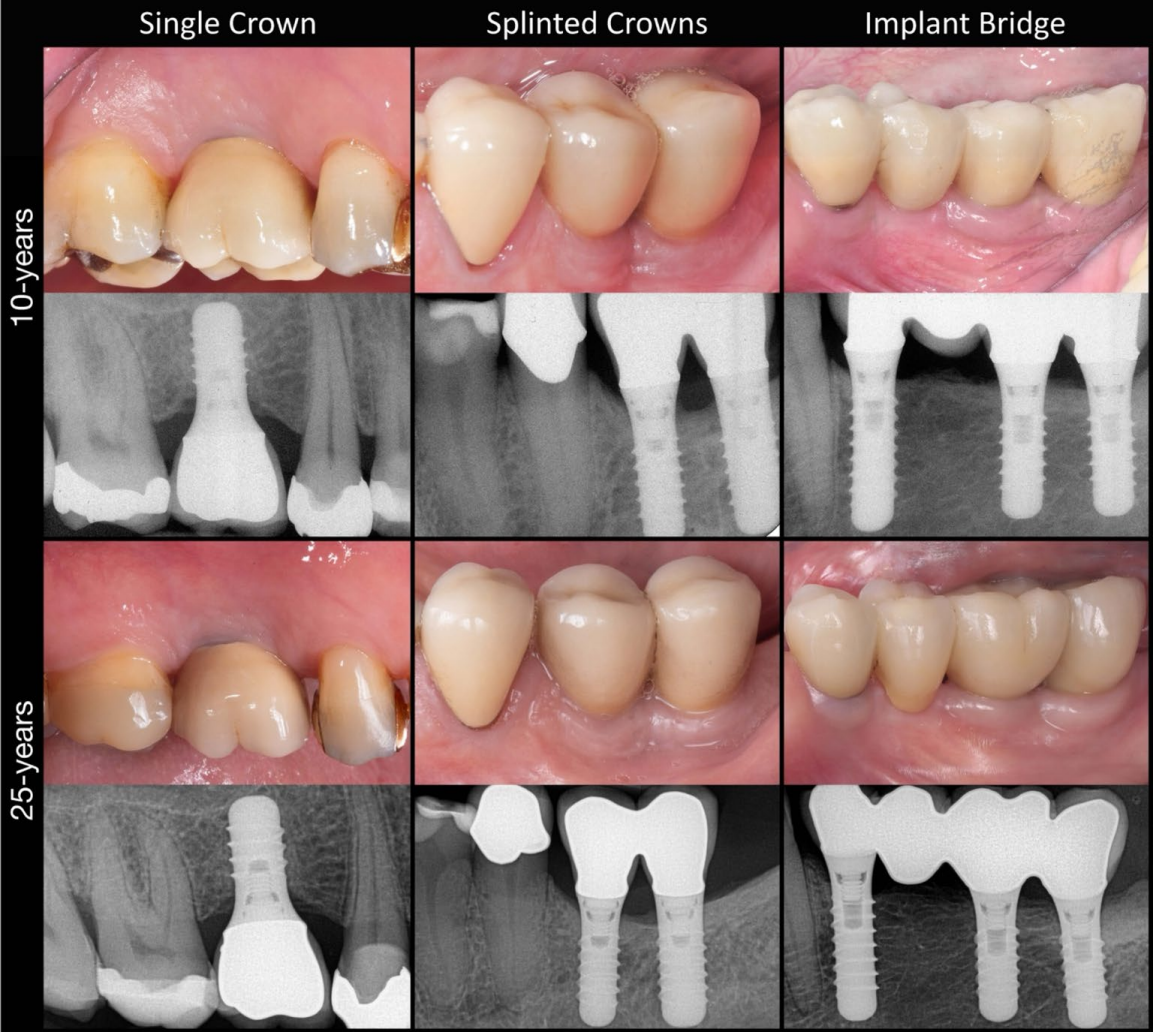
Clinical images and corresponding periapical radiographs of representative cases from each study group at the 10‐year and 25‐year follow‐up. Note that the implant bridge was redone due to chipping.

A total of 150 patients (82 women, 68 men; mean age 72.8 ± 13.1 years) with 237 implants were evaluated, while 15 implants had been lost (*n* = 9 loss of osseointegration, *n* = 6 peri‐implantitis) during the observation period and were excluded. The implants were evaluated first, after a mean of 10.6 ± 0.7 years, and second, after a mean of 25.1 ± 0.9 years postimplant placement. Patient age differed significantly between study groups, with 69 ± 13 years for SC, 76 ± 12 years for SP, and 79 ± 10 years for BR (*p* < 0.001). History of periodontal disease was reported by 31 patients. Regarding smoking status, 8 patients were light smokers (< 10 cigarettes/day) and 6 were heavy smokers. The corresponding IFDPs were SC (*n* = 129 reconstructions/*n* = 129 implants), SP (*n* = 20/40), SP with cantilever (*n* = 13/23), BR (*n* = 16/34), BR with cantilever (*n* = 4/7), tooth‐implant‐supported bridges (*n* = 1/1), or removable dental prostheses on single attachments (*n* = 3/3), with screw‐retention on *n* = 46 implants and cement‐retention on *n* = 191 implants. Due to small group sizes, tooth‐implant‐supported bridges and removable dental prostheses were excluded from further analysis (Figure 1).

The final sample comprised 147 patients with 233 implants, located in the maxilla (*n* = 95) and mandible (*n* = 138). BR were significantly more frequent in the maxilla compared with SC and SP (*p* = 0.005). No statistically significant differences were observed among IFDP types with respect to implant position (*p* = 0.14), implant length (*p* = 0.89), implant platform (*p* = 0.06), augmentation procedures (*p* = 0.12) or timing (*p* = 0.51), type of IFDP retention (*p* = 0.25), or marginal fit (*p* = 0.75). Nevertheless, implants with a 4.1 mm diameter and 2.8 mm platform height were most used for SP and BR, whereas 4.8 mm implants and 1.8 mm platform heights were more frequent for SC (*p* ≤ 0.03).

At 10 years, the prevalences of PH for SC, SP, and BR were 15.5%, 17.5%, and 22.0%, respectively; corresponding prevalences of PM were 82.2%, 81.0%, and 78.0%, and of PI were 2.3%, 1.6%, and 0%, respectively. At 25 years, PH was observed in 17.8%, 6.3%, and 14.6% of implants, PM in 74.4%, 87.3%, and 80.5%, and PI in 7.8%, 6.3%, and 4.9%. The prevalences between study groups did not differ significantly at both timepoints (*p* ≥ 0.95). Detailed characteristics are summarized in Table 1. The ICCs regarding CBL and emergence angle measurements ranged from 0.91 to 0.96 (95% CI: 0.89 to 0.97), indicating high inter‐rater reliability.

**TABLE 1:** Summary of demographic, implant‐, and site‐related characteristics and of clinical and radiographic data by type of implant‐supported prosthesis.

Characteristic	Single crowns *n* = 129	Splinted crowns *n* = 63	Bridges *n* = 41	*p*
Gender				0.97
Male	57 (44.2%)	31 (49.2%)	18 (43.9%)	
Female	72 (55.8%)	32 (50.8%)	23 (56.1%)	
Age at 25‐year follow‐up	69 [71] (13)	76 [81] (12)	79 [80] (10)	< 0.001*
Jaw				0.005
Maxilla	62 (48.1%)	28 (44.4%)	5 (12.2%)	
Mandibula	67 (51.9%)	35 (55.6%)	36 (87.8%)	
Implant position				0.14
Incisors	17 (13.2%)	1 (1.6%)	2 (4.9%)	
Canines	12 (9.3%)	4 (6.3%)	2 (4.9%)	
Premolars	43 (33.3%)	34 (54.0%)	19 (46.3%)	
Molars	57 (44.2%)	24 (38.1%)	18 (43.9%)	
Implant diameter				0.03*
3.3 mm	7 (5.4%)	0 (0.0%)	1 (2.4%)	
4.1 mm	55 (42.6%)	39 (61.9%)	29 (70.7%)	
4.8 mm	67 (51.9%)	24 (38.1%)	11 (26.8%)	
Implant length				0.89
≤ 10 mm	81 (62.8%)	42 (66.7%)	25 (61.0%)	
> 12 mm	48 (37.2%)	21 (33.3%)	16 (39.0%)	
Implant platform diameter				0.06
3.5 mm	7 (5.4%)	0 (0.0%)	1 (2.4%)	
4.8 mm	87 (67.4%)	62 (98.4%)	40 (97.6%)	
6.5 mm	35 (27.1%)	1 (1.6%)	0 (0.0%)	
Implant platform height				0.01*
1.8 mm	24 (18.6%)	2 (3.2%)	3 (7.3%)	
2.8 mm	105 (81.4%)	61 (96.8%)	38 (92.7%)	
Bone augmentation procedure				0.12
None	87 (67.4%)	44 (69.8%)	37 (90.2%)	
Guided bone regeneration	28 (21.7%)	8 (12.7%)	3 (7.3%)	
Sinus floor elevation	14 (10.9%)	11 (17.5%)	1 (2.4%)	
Bone augmentation timepoint				0.51
None	87 (67.4%)	44 (69.8%)	37 (90.2%)	
Simultaneous	25 (19.4%)	7 (11.1%)	4 (9.8%)	
Staged	17 (13.2%)	12 (19.0%)	0 (0.0%)	
IFDP type of retention				0.25
Cement‐retained	95 (73.6%)	55 (87.3%)	39 (95.1%)	
Screw‐retained	34 (26.4%)	8 (12.7%)	2 (4.9%)	
Prosthetic marginal fit				0.75
Adequate	115 (89.1%)	48 (76.2%)	35 (85.4%)	
Misfit	14 (10.9%)	15 (23.8%)	6 (14.6%)	
Peri‐implant tissue conditions at 10‐year follow‐up				0.99
Peri‐implantitis	3 (2.3%)	1 (1.6%)	0 (0.0%)	
Mucositis	106 (82.2%)	51 (81.0%)	32 (78.0%)	
Healthy	20 (15.5%)	11 (17.5%)	9 (22.0%)	
Peri‐implant tissue conditions at 25‐year follow‐up				0.95
Peri‐implantitis	10 (7.8%)	4 (6.3%)	2 (4.9%)	
Mucositis	96 (74.4%)	55 (87.3%)	33 (80.5%)	
Healthy	23 (17.8%)	4 (6.3%)	6 (14.6%)	
Clinical parameters				
Keratinized mucosa width	2.37 [2.00] (1.40)	2.03 [2.00] (1.25)	1.83 [2.00] (1.38)	0.14
Facial mucosal thickness	2.02 [2.00] (0.61)	2.09 [2.00] (0.67)	1.86 [2.00] (0.56)	0.34
Mean probing depths	3.07 [2.83] (1.20)	3.01 [2.67] (0.96)	2.96 [3.00] (1.15)	0.46
Bleeding on probing	0.42 [0.33] (0.26)	0.47 [0.50] (0.22)	0.36 [0.33] (0.14)	0.02*
0 Sites	17 (13.2%)	2 (3.2%)	1 (2.4%)	
1–2 Sites	55 (42.6%)	27 (42.9%)	29 (70.7%)	
3+ Sites	57 (44.2%)	34 (54.0%)	11 (26.8%)	
Suppuration on probing				0.50
No	122 (94.6%)	62 (98.4%)	41 (100.0%)	
Yes	7 (5.4%)	1 (1.6%)	0 (0.0%)	
Radiographic parameters				
Mean 10‐year CBL	0.23 [0.30] (0.83)	0.06 [0.10] (0.85)	0.13 [0.15] (0.68)	0.53
Mean 25‐year CBL	−0.14 [0.00] (1.29)	−0.31 [−0.05] (0.98)	−0.58 [−0.25] (1.16)	0.26
Mean ΔCBL	−0.37 [−0.15] (0.91)	−0.25 [−0.25] (0.85)	−0.70 [−0.45] (1.06)	0.37
Emergence angle mesial	23 [21] (11)	19 [18] (9)	21 [19] (10)	0.04*
Emergence angle distal	23 [21] (11)	22 [19] (10)	24 [23] (13)	0.98
Emergence profile level mesial				0.001*
Concave	17 (13.2%)	16 (25.4%)	17 (41.5%)	
Convex	67 (51.9%)	16 (25.4%)	8 (19.5%)	
Straight	45 (34.9%)	31 (49.2%)	16 (39.0%)	
Emergence profile level distal				0.007*
Concave	12 (9.4%)	18 (28.6%)	13 (31.7%)	
Convex	70 (54.7%)	21 (33.3%)	12 (29.3%)	
Straight	46 (35.9%)	24 (38.1%)	16 (39.0%)	
Anatomical crown/Implant ratio	0.76 [0.70] (0.24)	0.81 [0.80] (0.20)	0.74 [0.70] (0.21)	0.23

*Note:* Data presented as Mean [Median] (SD) for continuous and *n* (%) for count data.

Abbreviations: CBL, Crestal bone levels; IFDP, Implant‐supported fixed dental prosthesis. ΔCBL, Crestal bone level changes.

* Statistical significance.

### 3.2 ΔCBL According to IFDP Type, and Factors Associated With ΔCBL

**FIGURE 2:**
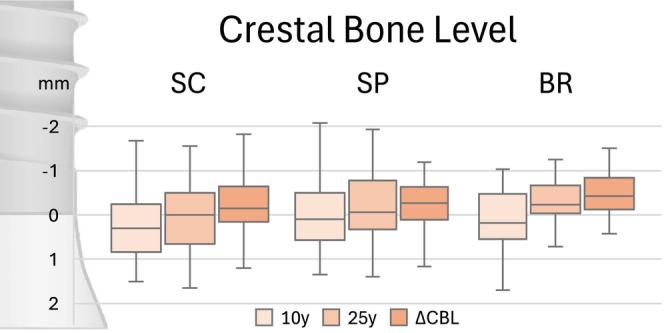
Boxplots of the 10‐ and 25‐year crestal bone levels, as well as the changes depending on the type of implant‐supported prosthesis. BR, Implant bridge; CBL, Crestal bone level; SC, Single implant crowns; SP, Splinted implant crowns.

Mean CBL at 10 versus 25 years were 0.23 ± 0.83 versus −0.14 ± 1.29 mm for SC, 0.06 ± 0.85 versus −0.31 ± 0.98 mm for SP, and 0.13 ± 0.68 versus −0.58 ± 1.16 mm for BR. The corresponding mean ΔCBL accounted for −0.37 ± 0.91 mm for SC, −0.25 ± 0.85 mm for SP, and −0.70 ± 1.0 mm for BR (Figure 2). No significant differences were observed among IFDP types for CBL at 10 years, 25 years, or for ΔCBL (*p* ≥ 0.26).

The mean transmucosal emergence angle was 23.0° ± 11.3° for SC, 20.4° ± 10.1° for SP, and 22.4° ± 13.3° for BR. SP demonstrated significantly narrower mesial emergence angles compared with SC and BR (*p* = 0.04).

Emergence profiles were classified as concave, convex, and straight in 11.0%, 53.7%, and 35.3% for SC; 27.0%, 29.4%, and 43.7% for SP; and 36.6%, 24.4%, and 39.0% for BR, respectively. Straight profiles were more frequent in the SP and BR groups, whereas the SC group exhibited a higher frequency of convex profiles (*p* ≤ 0.007).

Multivariate analyses showed that a significantly greater ΔCBL was associated with implant placement in incisor regions compared to canines (0.74 mm), premolars (0.78 mm), and molars (0.86 mm; *p* < 0.001). In addition, ΔCBL increased with greater probing depth (−0.16 mm per additional millimeter; *p* < 0.001) and was markedly higher in the presence of SOP (−0.97 mm; *p* < 0.001). However, no significant associations were found between ΔCBL and IFDP type, platform height, transmucosal emergence geometry, and KMW or FMT. Detailed results of the multivariate analysis regarding ΔCBL are presented in Table 2.

**TABLE 2:** Multivariate analysis of risk factors for crestal bone level changes over time.

	Effect	*p*
Intercept	0.825	
IFPD		0.17
Single crowns	Baseline	
Splinted crowns	+0.124	
Bridges	+0.198	
Platform height		0.17
2.8 mm	Baseline	
1.8 mm	+0.150	
Position		< 0.001*
Incisors	Baseline	
Canines	−0.739	
Premolars	−0.784	
Molars	−0.855	
Augmentation		0.10
Probing depths	+0.157	< 0.001*
Bleeding on probing		0.26
0 Sites	Baseline	
1–2 Sites	+0.127	
3+ Sites	+0.038	
Suppuration on probing		< 0.001*
No	Baseline	
Yes	+0.968	
Keratinized mucosa width	+0.013	0.61
Facial mucosal thickness	+0.026	0.67

Abbreviation: IFDP, Implant‐supported fixed dental prosthesis.

* Statistical significance.

### 3.3 Additional Radiographic Findings

**FIGURE 3:**
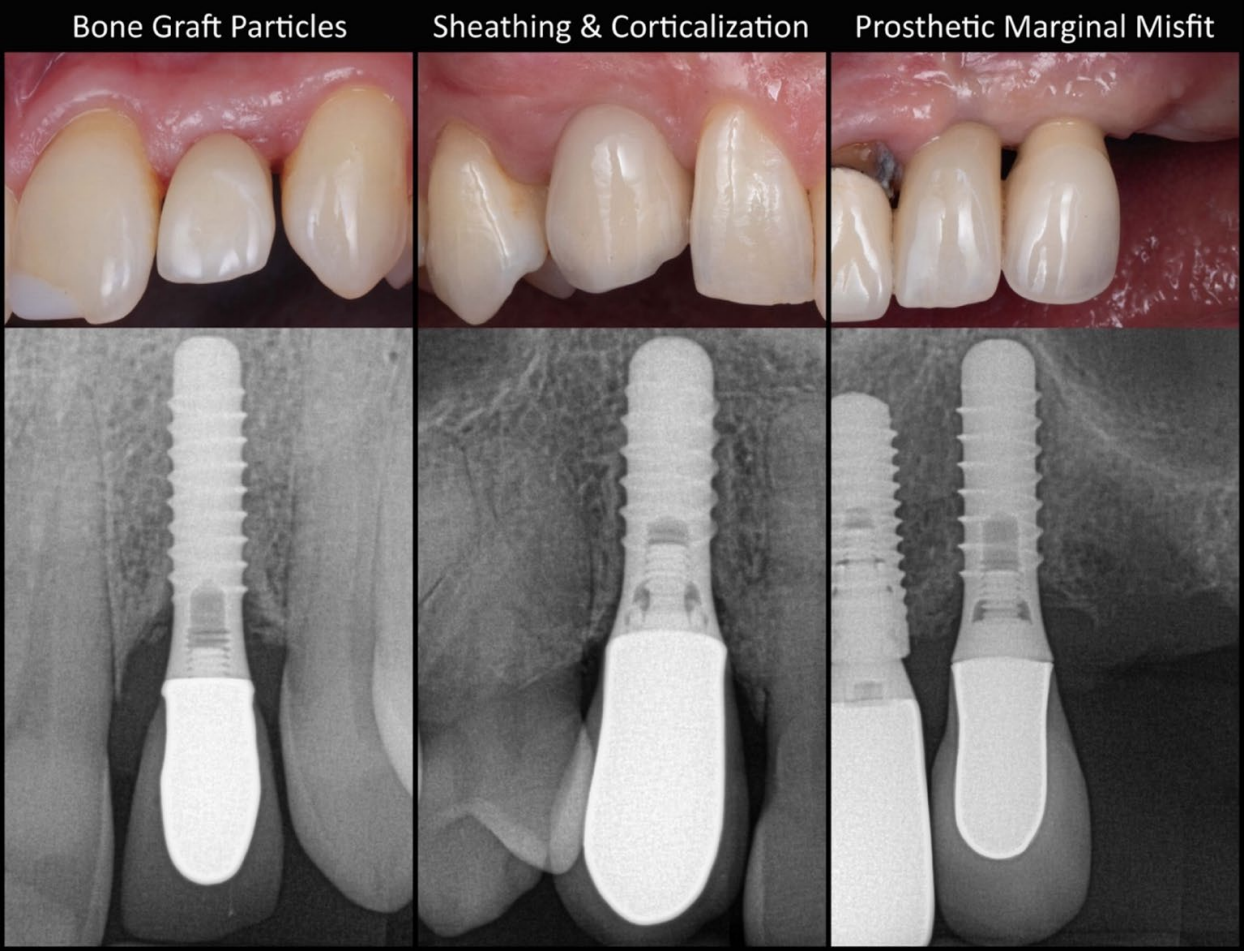
Clinical images and corresponding periapical radiographs of representative cases with adverse radiographic findings.

Residual bone grafting material was detected in 8.5% for SC, 9.5% for splinted crowns SP, and 0% for BR. Peri‐implant sheathing and corticalization were observed in 10.1% and 20.9% for SC, 12.7% and 12.7% for SP, and 9.8% and 19.5% for BR, respectively. When present, peri‐implant sheathing was predominantly associated with a CBL < 3 mm (80%), whereas the remaining cases presented a CBL ≥ 3 mm (20%). A prosthetic misfit of the IFDP was identified in 10.9% for SC, 23.8% for SP, and 14.6% for BR. However, no significant associations were found between prosthetic misfit and PH in this tissue‐level cohort. No radiographic signs of residual cement, retrograde PI, or implant fracture were observed. Representative cases and details on the subgroup prevalence of radiographic findings are displayed in Figure 3 and Table 3.

**TABLE 3:** Prevalences of radiographic findings depending on the type of implant‐supported fixed dental prosthesis.

	Single crowns *n* = 129		Splinted crowns *n* = 63		Bridges *n* = 41	
	*n*	%	*n*	%	*n*	%
Isolated bone graft material	11	8.5	6	9.5	0	0
Peri‐implant sheathing	13	10.1	8	12.7	4	9.8
Peri‐implant corticalization	27	20.9	8	12.7	8	19.5
Prosthetic marginal misfit	14	10.9	15	23.8	8	19.5
Cement remnants	0	0	0	0	0	0
Implant fracture	0	0	0	0	0	0
Retrograde peri‐implantitis	0	0	0	0	0	0

## 4 Discussion

### 4.1 Main Findings

This observational study assessed the influence of IFDP type on long‐term ΔCBL, and PM and PI rates at tissue‐level implants with a hybrid surface design. IFDP type was not significantly associated with ΔCBL or the PM and PI rates. In contrast, several implant‐ and site‐related factors were significantly associated with greater ΔCBL, including implant placement in incisor regions, increased PD, and SOP.

### 4.2 Agreements and Disagreements With Existing Evidence

In the present study, 10‐ and 25‐year prevalences of PM and PI did not significantly differ among IFDP types, although a trend toward higher PM prevalence was observed for SP compared with SC and BR at 25 years. This is contradictory to a long‐term retrospective study with 17–23 years of follow‐up, which reported higher PM and PI prevalences for implant‐supported fixed partial dentures compared to SC [11]. PM has also been associated with short‐span IFDPs [33], and higher prevalences of PI have been observed for SP compared to SC or BR [34], and for BR compared to SC [35]. The latter might be attributed to reduced accessibility for oral hygiene measures in multi‐unit reconstructions, especially if compromises in IFDP designs are present [35, 36]. Conversely, a recent meta‐analysis found no effect of splinting on biological complication rates in posterior adjacent implants [37]. Comparisons with the literature are therefore hampered by heterogeneous definitions and groupings of IFDP types, as well as differences in study design and follow‐up duration. Greater methodological standardization in future studies may help clarify these inconsistencies. Nevertheless, the present findings suggest that, over extended observation periods and for well‐designed prostheses on tissue‐level implants, the type of IFDP may play a limited role in long‐term peri‐implant tissue stability compared with patient‐ and site‐related factors.

Interestingly, CBL remained largely stable over the 25‐year observation period, with only slightly greater ΔCBL observed for BR as compared to SP and SC. However, neither the 10 nor the 25‐year CBL nor ΔCBL differed significantly between the different types of IFDPs. This finding contrasts with a recent systematic review and meta‐analysis that identified splinting as a risk factor for crestal bone loss [12]. However, other systematic reviews did not find a significant difference in crestal bone loss between splinted and nonsplinted adjacent implants, suggesting that the effect may be modest and context‐dependent if prosthesis design is appropriate and cleansable [38].

Concerning the prosthetic emergence geometry, neither emergence angles nor profiles were associated with increased ΔCBL. This observation aligns with two previous studies, which did not find an association between emergence geometry and peri‐implant tissue conditions at TL implant design [20, 21]. In contrast, a recent study identified an emergence angle of 48° in tissue‐level implant–supported prostheses as a critical threshold for the development of PM and PI, suggesting that overly steep emergence profiles have been implicated in plaque accumulation and peri‐implant inflammation [22]. Hence, the present findings underscore the emerging evidence on the potential of tissue‐level implants with a machined implant surface in the supracrestal area to compensate for prosthetic emergence compromises concerning CBL, PM, and PI prevalences. Nevertheless, excessively steep emergence profiles may still constitute a local contributing factor for biological complications, particularly in the presence of inadequate plaque control.

Interestingly, prosthetic misfit was not associated with ΔCBL. This finding is consistent with the results of a recent systematic review, which failed to identify a clinical threshold of an acceptable misfit [39]. The absence of an observed effect may be partly related to the tissue‐level implant designs in the present cohort, with most implants featuring a 2.8 mm platform height, thereby displacing the critical implant–abutment microgap away from the crestal bone. Increasing the vertical distance between the bone crest and the prosthetic microgap may limit the apical extension of the inflammatory infiltrate, promote a more effective sealing of the supracrestal soft tissues, and reduce both microbial‐ and load‐induced stress at the crestal bone level [40, 41, 42, 43]. Consequently, even in the presence of marginal misfit at the implant–superstructure interface, this configuration may mitigate the initiation of peri‐implant inflammation and reduce the progression of crestal bone loss [10, 36, 43].

Implant location emerged as a strong determinant of ΔCBL, with implants placed in anterior regions exhibiting significantly greater ΔCBL compared with those placed in nonincisor sites. This observation is consistent with a previous retrospective study reporting a 3.7‐fold higher probability of bone loss for anterior compared with posterior sites [44]. In addition, meta‐analyses have demonstrated a significantly increased risk of PI in both maxillary and mandibular anterior regions, with pooled risk ratios ranging from 1.34 to 1.76 compared with posterior sites [45]. These site‐related differences may be attributed to the typically thinner hard‐ and soft‐tissue phenotypes in the anterior region, which may be more susceptible to remodeling and inflammatory breakdown. In addition, greater nonaxial loading associated with incisor inclination during function may contribute to increased biomechanical stress at the crestal bone, thereby promoting greater ΔCBL over time [46].

Clinical inflammatory parameters showed strong associations with ΔCBL. In the presence of SOP, the mean ΔCBL increased by −0.97 mm, while for each additional millimeter of PD, ΔCBL increased by −0.16 mm. In contrast, no significant association was identified for BOP. Recent evidence indicates high specificity for the diagnosis of recent peri‐implant bone loss for SOP, PD ≥ 6 mm, or increases in PD over time ([47, 48, 49]). SOP, in particular, has been associated with a distinct and more pathogenic peri‐implant microbiome compared with sites without SOP and likely reflects advanced inflammatory tissue breakdown, explaining its strong association with crestal bone loss [50]. Although implants with a recent history of bone loss generally present with BOP, the predictive and diagnostic value of detecting one or two BOP sites is limited due to their low specificity [49]. However, probing measurement accuracy around implants may be affected by prosthetic design. A recent study showed that prosthesis removal improved measurement accuracy, while peri‐implantitis, concave restorative profiles, and anterior implant position contributed to increased probing discrepancies [51, 52]. Collectively, these observations underscore the central role of peri‐implant inflammation in crestal bone remodeling and align with the established pathophysiology of PM and PI [53, 54]. These further highlight the importance of regular supportive peri‐implant care, early detection of inflammatory changes, and timely therapeutic intervention to preserve peri‐implant bone stability [55]. This is especially relevant because current treatment options are often unpredictable in achieving complete disease resolution, regardless of peri‐implant supportive care following PM and PI treatment [56].

In specific cases, crestal bone loss around IFDPs may also appear in the absence of clinical symptoms of inflammation. Radiographically, this observation is often characterized by a linear peri‐implant radiolucency in the crestal area (i.e., sheathing), sometimes with concomitant corticalization. This pattern has been suggested to reflect aseptic disruption of osseointegration at the bone–implant interface, a process that is not yet fully understood [57]. Proposed mechanisms include aseptic immune‐mediated responses to the titanium particles, potentially leading to rapid bone loss [3]. Others suggest postloading peri‐implant bone densification, possibly related to functional adaptation or occlusal trauma, may compromise local blood supply and promote the formation of a soft tissue interface along part or the entire implant surface [31, 58]. Such phenomena might vary with different prosthetic configurations, as splinting aims to distribute occlusal loads and reduce the risk of implant overloading [59]. However, in the present analysis, peri‐implant sheathing was observed in 10.1%–14.6% of implants, and was not influenced by the type of IFDP. It is important to acknowledge that, to date, no consensus has yet been established regarding the etiology or clinical management of these radiographic peri‐implant findings.

### 4.3 Limitations

The present long‐term study has several limitations. First, the observational design and the single‐center academic setting may limit the generalizability of the findings to other clinical environments, practitioners, or patient populations, and do not allow causal inferences to be drawn. Second, only available patients who consented and had sufficient clinical and radiographic documentation at both the 10‐ and 25‐year follow‐ups were included, potentially affected by positive selection bias and leading to an underestimation of the extent of CBL changes and the prevalence of PM and PI. Third, although IFDP categories were predefined, heterogeneity within groups (e.g., splinted vs. nonsplinted reconstructions, presence of cantilevers, and retention type) may have diluted potential associations. Fourth, CBL and emergence geometry were assessed using panoramic radiographs to minimize angulation effects. Panoramic imaging, however, is subject to distortion and lower spatial resolution compared with intraoral radiographs. In addition, the two‐dimensional radiographic assessment does not capture buccal or lingual bone changes, which may be clinically relevant. Additionally, it should be acknowledged that the systemic risk factors reported refer to the 25‐year follow‐up and may not reflect the patients' status at baseline or at earlier time points. As systemic risk factors can change over the course of a long‐term observation period, caution is warranted when interpreting their association with peri‐implant outcomes.

Future well‐designed prospective cohort studies and randomized clinical trials are needed to confirm whether the observed limited influence of IFDP type on early CBL and the onset of PM and PI persists across different clinical settings, implant systems, and patient populations. Multicenter designs would enhance external validity and allow assessment of operator‐ and center‐related effects.

## 5 Conclusions

Within the limitations of the present long‐term analysis of tissue‐level implants, it can be concluded that:

− Crestal bone level changes (ΔCBL) remained minimal over the observation of 25‐years and were not influenced by the implant‐supported prosthesis (IFDP) type.
− The prevalence of peri‐implant mucositis and peri‐implantitis at 10‐ and 25‐year was not associated with IFDP type.
− Implant placement in anterior regions was significantly associated with greater ΔCBL.
− Suppuration on probing and increased probing depths were significantly associated with greater ΔCBL.
− Prosthetic emergence geometry did not influence ΔCBL.

## Author Contributions

C.R. and E.C.‐Q. conceived the idea and designed the study. C.R., M.F., A.R., G.E.S., and E.C.‐Q. acquired and analyzed the data. C.R. led the writing. C.R., M.F., A.R., M.S., G.E.S., V.C., and E.C.‐Q. contributed to data interpretation and critically revised the manuscript. All authors gave final approval and agreed to be accountable for all aspects of the scientific work.

## Funding

This study was supported by a research grant from the International Team for Implantology (ITI) (Grant No. 1574‐2021).

## Ethics Statement

The study received approval from the Standing Ethics Committee for Clinical Studies of the State of Bern, Switzerland (KEK‐BE No. 2023‐02279).

## Conflicts of Interest

The authors declare no conflicts of interest.

## Data Availability

The data that support the findings of this study are available on request from the corresponding author. The data are not publicly available due to privacy or ethical restrictions.
